# HLA-DR3 restricted environmental epitopes from the bacterium *Clostridium tetani* have T cell cross-reactivity to the SLE-related autoantigen SmD

**DOI:** 10.3389/fimmu.2022.928374

**Published:** 2022-10-31

**Authors:** Zhenhuan Zhao, Ashley N. Anderson, Carol C. Kannapell, William W. Kwok, Felicia Gaskin, Shu Man Fu

**Affiliations:** ^1^ Division of Rheumatology, Department of Medicine, University of Virginia, Charlottesville, VA, United States; ^2^ Center for Immunity, Inflammation and Regenerative Medicine, Division of Nephrology, Department of Medicine, University of Virginia, Charlottesville, VA, United States; ^3^ Benaroya Research Institute, Virginia Mason, Seattle, WA, United States; ^4^ Department of Psychiatry and Neurobehavioral Sciences, University of Virginia, Charlottesville, VA, United States

**Keywords:** T cell epitope, HLA-DR3, environmental bacteria, molecular mimicry, autoantigen, Smith D (SmD), cross-reactive T cells, systemic lupus erythematosus (SLE)

## Abstract

HLA-DR3 (DR3) is one of the dominant HLA-DR alleles associated with systemic lupus erythematosus (SLE) susceptibility. Our previous studies showed multiple intramolecular DR3 restricted T cell epitopes in the Smith D (SmD) protein, from which we generated a non-homologous, bacterial epitope mimics library. From this library we identified ABC_247-261_ Mimic as one new DR3 restricted bacterial T cell epitope from the ABC transporter ATP-binding protein in *Clostridium tetani*. It activated and induced autoreactive SmD_66-80_-specific T cells and induced autoantibodies to lupus-related autoantigens *in vivo*. Compared to healthy donors, SLE patients have a greater percentage of cross-reactive T cells to ABC_247-261_ Mimic and SmD_66-80_. In addition, we analyzed the ability of single DR3 restricted Tetanus toxoid (TT) T cell epitopes to induce autoimmune T cells. We found that the immunodominant TT epitope TT_826-845_ stimulated SmD_66-80_ reactive T cells but failed to induce persistent anti-SmD autoantibodies compared to the ABC_247-261_ Mimic. Thus, exposure to the ABC_247-261_ Mimic epitope may contribute to autoimmunity in susceptible DR3 individuals.

## Introduction

Systemic lupus erythematosus (SLE) is a prototypical, systemic autoimmune disease that occurs when the body’s immune system begins to attack its own healthy tissues (loss of self-tolerance) (1). Autoantibodies are one of the clinical markers of lupus and can be detected prior to symptoms and diagnosis (2). There is no cure for SLE and the exact mechanisms underlying disease development have yet to be elucidated.

The HLA-DR region is the dominant lupus susceptibility locus, particularly the HLA-DR3 (DR3) allele. This highlights the importance of T cells in lupus because the genes within the HLA-DR locus are important for antigen presentation to T cells (3–5). It is known that environmental antigens that share T cell epitopes with self-antigens can initiate adaptive immune responses that continue to be propagated by self-antigens, leading to disease in genetically susceptible individuals (6). Our laboratory has shown that in DR3 transgenic mice (DR3 mice), T cells specific for SLE-related autoantigens responded to bacterial mimics of self-peptides. For example, DR3 restricted T cells specific for SmD (7), and Ro60 (8) responded to several epitopes from environmental bacteria. We also identified numerous potential bacterial mimics that were able to induce the production of SLE-related autoantibodies in DR3 mice (9). However, the existence of cross-reactive T cells able to recognize self and non-homologous bacterial epitopes in humans remains to be demonstrated. Furthermore, if these cross-reactive T cells are present in humans, it is of interest whether they are activated by bacterial epitopes to induce lupus-related autoantibodies.

In this study, we examined the epitopes from SmD and the bacterium *Clostridium tetani* for the presence of shared T cell epitopes. To do this, we utilized MHC class II DR3 tetramers carrying different epitopes. Our results show that one bacterial epitope from the ABC transporter ATP-binding protein (ABC_247-261_ Mimic) activated SmD_66-80_ reactive T cells in both DR3 mice and humans. The percentages of cross-reactive T cells to ABC_247-261_ Mimic and SmD_66-80_ were significantly increased in DR3 positive SLE patients (DR3 SLE patients). Additionally, ABC_247-261_ Mimic induced persistent anti-SmD autoantibodies in DR3 mice. Our results suggest that environmental mimics of self-epitopes may be important for activating cross-reactive T cells, thus potentially leading to the initiation of autoimmune responses and SLE development in susceptible individuals.

## Materials and methods

### Human subjects

Patients from the University of Virginia Hospital fulfilling the American College of Rheumatology (ACR) criteria for systemic lupus erythematosus (SLE) (10) and healthy donors provided blood samples for HLA-DR typing. Patients’ and donors’ samples were archived and deidentified. Only HLA-DR3 (DR3) individuals were selected for this study. The three selected patients are two African American females and one Caucasian female over the age of 18. The three patients were all ANA and anti-SmD positive with leukopenia, oral mucosal and joint involvement. The patients were also anti-Tetanus toxoid (TT) positive [Mean, 0.81 ± 0.20] at 1 to 800 dilution. Seven of eight healthy donors were anti-TT positive [Mean, 0.96 ± 0.65] at 1 to 800 dilution (p=0.57).

### Isolation of human peripheral blood mononuclear cells from whole blood

Peripheral blood mononuclear cells (PBMCs) were isolated by standard Ficoll-Paque PLUS (GE Healthcare, Chicago, IL) gradient centrifugation, resuspended in freezing media (10% DMSO in FCS), and stored in liquid nitrogen.

### HLA-DR typing

DNA was extracted from blood clots and HLA-DR typing was done by the PCR method described by Bunce (11).

### Stimulation and DR3 tetramer staining of mouse spleen cells and human PBMCs

This procedure was performed using modified versions of protocols described previously (12, 13). Briefly, human PBMCs were stimulated with 17.7 μg/ml of TT lysate (MassBiologics, University of Massachusetts Medical School, MA) for 5-7 days. Spleen cells from DR3 mice immunized with protein or peptide were harvested 10-14 days post immunization. Human PBMCs were stained with 1 μg of DR3 tetramer for 3-4 hours at 37°C; and mouse spleen cells were stained for 2 hours. After tetramer staining, the cells were washed in PBS containing 2% FCS (FACS buffer) and surface markers were detected by anti-CD4-FITC (Biolegend, San Diego, CA) and anti-CD3-APCeFluor™780 (Invitrogen, Waltham, MA) for human cells and anti-CD4-FITC and anti-CD3-APC/Fire™750 (Biolegend, San Diego, CA) for mouse cells on ice for 30 minutes after Fc blocking. Cells were analyzed by the BD LSR Fortessa™ Cell Analyzer (BD, Franklin Lakes, NJ). When determining the percentage of single epitope reactive T cells from dual tetramer staining as seen in **Figure 6**, for SmD_66-80_, the percentage of single SmD_66-80_ tetramer positive T cells was the sum of cells in Q1 and Q2; for the ABC_247-261_ Mimic and TT peptides, the percentage of single *Clostridium* tetani epitope tetramer positive T cells was the sum of cells in Q2 and Q3. Cross-reactive T cells between SmD_66-80_ and *Clostridium* tetani epitopes were detected in Q2.

### Peptides and recombinant proteins

DR3 restricted TT peptides TT_92-111_, TT_279-296_, TT_826-845_, TT_986-1005_, TT_1058-1077_, TT_1114-1133_, and TT_1272-1284_, as well as the bacterial ABC_247-261_ Mimic peptide and peptides TT_31-45_ and TT_506-525_ were HPLC purified with ≥95% purity and obtained from GenScript (Piscataway, New Jersey). The overlapping, 30 amino acid length SmD peptides spanning the entire protein (119 amino acids), as well as 15mers SmD_1-15_ and SmD_66-80_ were also HPLC purified with ≥95% purity and obtained from ChinaPeptides (Shanghai, China). The recombinant SLE-related autoantigens used were described previously (14, 15). TT lysate was obtained from MassBiologics at the University of Massachusetts Medical School (UMMS). It was prepared from inactivated bacterial cultures followed by filtration and does not contain adjuvant.

### Mouse strains and immunizations

The HLA-DRB1*0301, HLA-DRA1*0101 (DR3.A^0/0^E^0/0^) transgenic mice lacking endogenous class II molecules have been previously described (16). The HLA class II transgenic mice used in the present work were backcrossed to B10 mice for 10 generations, and thus carry B10 genetic backgrounds. All mice used in this study were bred and housed in specific pathogen-free conditions at the University of Virginia vivarium. Mouse experiments were approved by the Animal Care and Use Committees at the University of Virginia. For experiments analyzing T cell IFN-γ responses, two mice were immunized in the left footpad and base of tail with 100 μg of peptide or protein in IFA. For experiments analyzing antibody production, 4-5 mice were immunized in the left footpad and base of tail with 100 μg of peptide in CFA. On days 14 and 28 post immunization, mice received injections of 50 μg of peptide in IFA intraperitoneally. Sera were collected monthly and stored at -20°C.

### T cell peptide-specific IFN-γ responses

For determining T cell specific IFN-γ responses to SmD_66-80_, ABC_247-261_ Mimic, and other TT peptides, two mice were immunized with each single peptide in the footpad and base of the tail, respectively and 10-14 days later spleen cells were pooled and analyzed. Cells were plated in 96-well round bottom plates at 2 x 10^6^ cells per well with 20 µg/ml peptide at 37°C in 5% CO_2_ for 2 days. Culture supernatants were used to determine IFN-γ concentrations by ELISA (BD, Franklin Lakes, NJ), following the manufacturer’s instructions.

### T-T hybridoma production and reactivity

SmD_66-80_ reactive T-T hybridomas were produced from the fusion of lymph node cells from DR3.A^0/0^E^0/0^ mice immunized with SmD in IFA with BW5147TCR^-/-^ as described by Kruisbeek (17). For each fusion, cells were suspended in ten 96-well plates. Hybrid cells surviving hypoxanthine/aminopterin/thymidine (HAT) selection were expanded in 24-well plates. 10^5^ T-T hybridoma cells were incubated with 2.5×10^5^ syngeneic splenic cells in the presence of SmD_66-80_ for 14 hours. Interleukin (IL)-2 in culture supernatants was estimated by ELISA (BD, Franklin Lakes, NJ) following manufacturer’s instructions. Reactive hybridomas were cloned by limiting dilution. To determine the ability of the T-T hybridoma to respond to TT, hybridoma cells were incubated with irradiated syngeneic spleen cells in the presence of TT and IL-2 in culture supernatants was measured by ELISA.

### Enzyme-linked immunosorbent assay

The experiments were performed using a modified version of the protocol described previously (14). Immulon 4HBX microtiter plates (ThermoFisher Scientific, Waltham, MA) were coated with 2 µg/ml of peptides overnight at 4°C and blocked with 1x PBS containing 0.1% Tween 20 with 3% BSA (blocking buffer) for 1 hour at room temperature. Mouse sera diluted 1 to 100 in blocking buffer were added to the plate and incubated for 2 hours. Secondary anti-mouse IgG in blocking buffer was added and incubated for 1hour. Then the substrate o-phenylenediamine (0.05%) (Sigma-Aldrich, St. Louis, MO) and 0.06% hydrogen peroxide in citrate-phosphate buffer, pH 5.0 (100 µl/well) was applied. Reaction was stopped after 15 minutes by the addition of 50 µl/well of 2.5 N sulfuric acid. Absorbance was read at 490 nm with a reference wavelength of 560 nm.

### Antibody absorption

Peptide SmD_66-80_ was coupled to CNBr-activated Sepharose 4B beads (GE Healthcare, Chicago, IL) following the manufacturer’s instructions. The beads were incubated overnight with PBS containing 3% BSA at 4°C. Sera were diluted in 1x PBS with 0.1% Tween 20 (PBST) containing 3% BSA and incubated with the beads for 2 hours at room temperature. The absorbed sera were assayed for anti-SmD_66-80_ activity using ELISA.

### Statistical analysis

Significance was determined using the two tailed Student’s t-test.

## Results

### TT lysate activates SmD_66-80_ cross-reactive T cells

**Figure 1:**
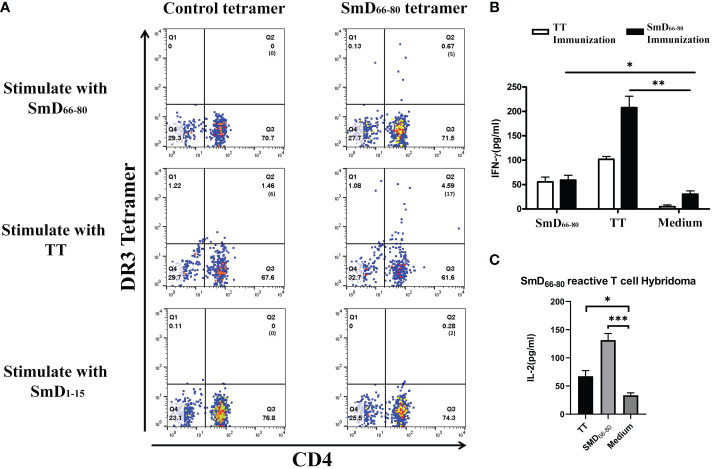
TT lysate activates autoimmune SmD_66-80_ reactive T cells. **(A)** The PBMCs from an SLE patient were stained with the SmD_66-80_ DR3 tetramer after stimulation with SmD_66-80_ or TT lysate. SmD_1-15_ was used as a negative control. The number of tetramer positive cells is shown in parentheses. **(B)** Humanized DR3 mice were immunized with TT (n=2) or SmD_66-80_ (n=2), respectively. The splenic T cell responses were recalled using SmD_66-80_ or TT. Supernatant IFN-γ was assayed for T cell activation. **(C)** T cell hybridoma F140 was previously shown to react to SmD_66-80_ (9). Its cross-reactivity to TT was assayed by stimulating it with TT. Supernatant IL-2 was assayed for hybridoma activation. *p < 0.05, **p < 0.01, ***p < 0.001 by Student’s two-tailed t-test. TT, Tetanus toxoid.

We previously identified SmD_66-80_ as one of seven DR3 restricted SmD T cell epitopes using T-T hybridomas in our DR3 transgenic mouse model. Many of the T-T hybridomas have cross-reactivity to SmD_66-80_ and bacterial antigens (9). Experiments were designed to demonstrate cross-reactivity to SmD_66-80_ and bacterial antigens on a single T cell by the HLA-DR3 tetramer technology. First to confirm the specificity of the SmD_66-80_ DR3 tetramer, DR3 mice were immunized with SmD_66-80_ and SmD_66-80_ reactive T cells were detected using the SmD_66-80_ DR3 tetramer. DR3 mice immunized with SmD_91-119_ were used as controls. SmD_91-119_ lacks the DR3 restricted T cell epitopes. 6.23% SmD_66-80_ reactive CD4^+^ T cells were identified in the spleens of SmD_66-80_ immunized DR3 mice. In comparison, 1.24% CD4^+^ T cells were seen in the spleens of SmD_91-119_ immunized DR3 mice. The control DR3 tetramer loaded with non SmD peptides showed 0.27% and 0.11% staining in immunized mice, respectively (**Supplementary Figure 1**). These results showed the SmD_66-80_ DR3 tetramer specifically bound SmD_66-80_ reactive CD4^+^ T cells. After establishing the specificity of the SmD_66-80_ DR3 tetramer, it was used to stain PBMCs from one SLE patient. The PBMCs were stimulated with the autoantigen peptide SmD_66-80_, and TT lysate (a strong T cell dependent antigen) was used as a negative control. We showed in one DR3 positive SLE patient that 0.67% of the CD4^+^ T cells were stained with the SmD_66-80_ DR3 tetramer. Surprisingly, the TT lysate stimulation increased the SmD_66-80_ reactive T cells to 4.59% (**Figure 1A**). These results suggested that there are T cell epitopes in the TT lysate that are cross-reactive with SmD_66-80_. To seek further support of this thesis, we immunized humanized DR3 mice with SmD_66-80_ or TT, respectively. We found that the peptides from either SmD or TT were able to stimulate the splenic T cells of the other (**Figure 1B**). This showed that TT immunization activates SmD_66-80_ reactive T cells and vice versa. Furthermore, a DR3 restricted SmD_66-80_ reactive T cell hybridoma generated from SmD immunization of DR3 mice was activated by TT (**Figure 1C**). These results suggested one or more DR3 restricted T cell epitopes in TT lysate activated and expanded autoimmune SmD_66-80_ reactive CD4^+^ T cells.

### An epitope from our bacterial mimicry library shows both T and B cell cross-reactivity to the SmD_66-80_ epitope

**Figure 2:**
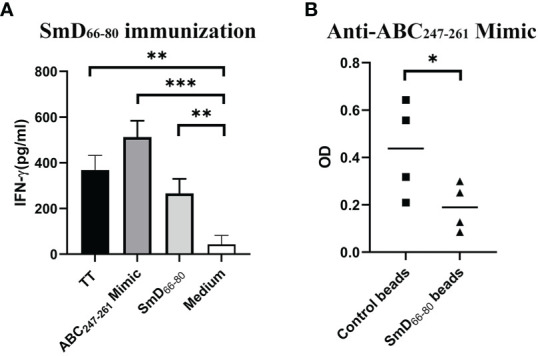
The ABC_247-261_ Mimic, one epitope from the bacterium *Clostridium tetani*, cross-reacts with SmD_66-80_ at both the T and B cell levels. **(A)** Splenic cells from two SmD_66-80_ immunized DR3 mice were stimulated with the ABC_247-261_ Mimic and SmD_66-80_ and TT lysate were used as positive controls. IFN-γ was assayed for T cell activation. **(B)** Sera from SmD_66-80_ immunized DR3 mice (n=4) were probed for ABC_247-261_ Mimic antibodies. SmD_66-80_ coated beads were used to absorb the cross-reactive antibodies between SmD_66-80_ and the ABC_247-261_ Mimic. *p < 0.05, **p < 0.01, ***p < 0.001 by Student’s two-tailed t-test. TT, Tetanus toxoid.

As reported previously (9), a software program was developed to analyze bacterial protein sequences in order to create a library of mimic peptides that may stimulate SmD reactive T cells. From this library, we identified one epitope (the ABC_247-261_ Mimic) from the bacterium *Clostridium tetani*, to have cross-reactivity with SmD. It is of note that this bacterium produces TT. We showed this epitope could activate T cells from DR3 mice primed by SmD_66-80_ (**Figure 2A**). At the B cell level, the DR3 mice immunized with SmD_66-80_ produced anti-SmD antibodies with cross-reactivity to ABC_247-261_ Mimic. We showed anti-ABC_247-261_ Mimic antibodies could be generated by SmD_66-80_ immunization of DR3 mice and could be absorbed by SmD_66-80_ coated beads (**Figure 2B**). These results suggest at least one bacterial epitope from our mimicry library has both T and B cell cross-reactivity to the SmD_66-80_ autoimmune response.

### ABC_247-261_ Mimic and TT T cell epitopes induce cross-reactive T cells against SmD and TT

**Figure 3:**
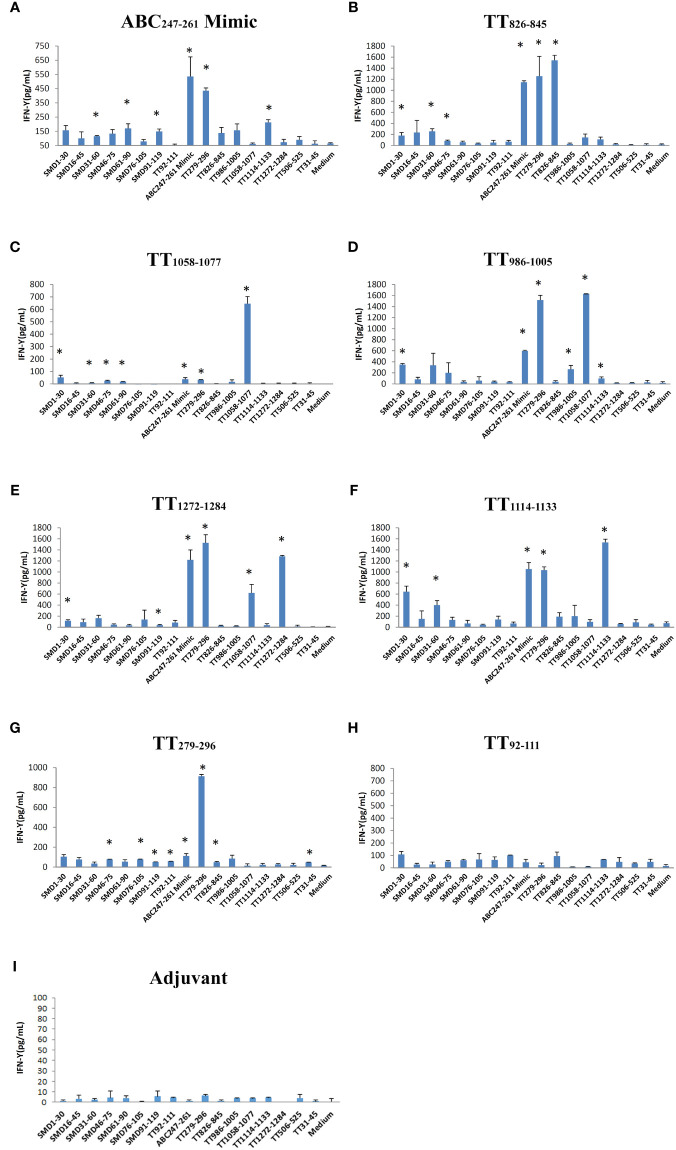
The ABC_247-261_ Mimic and single DR3 restricted TT epitopes induce cross-reactive T cells to SmD and TT epitopes. Peptides **(A)** the ABC_247-261_ Mimic, **(B)** TT_826-845_, **(C)** TT_1058-1077_, **(D)** TT_986-1005_, **(E)** TT_1272-1284_, **(F)** TT_1114-1133_, **(G)** TT_279-296_, and **(H)** TT_92-111_ were used to immunize DR3 mice. DR3 mice immunized with **(I)** adjuvant only were used as controls. Splenic cells from two immunized mice were stimulated with a panel of both SmD and TT epitope peptides. Supernatant IFN-γ was assayed for T cell activation. This experiment was replicated twice. * means the stimulating epitope peptide significantly increased the IFN-γ present in the supernatant compared to the medium only as indicated by Student’s two-tailed t-tests.

Seven DR3 restricted TT peptides have been described in the literatures (18–20). They are TT_92-111_, TT_279-296_, TT_826-845_, TT_986-1005_, TT_1058-1077_, TT_1114-1133_, and TT_1272-1284_ (**Table 1**). DR3 mice were immunized with individual TT peptides and ABC_247-261_ Mimic in incomplete Freund’s adjuvant (IFA). As a negative control, IFA was used (**Figure 3I**). Splenic T cells were cultured with the ABC_247-261_ Mimic, TT peptides and SmD peptides as recall antigens. The supernatants were analyzed for IFN-γ (**Figures 3A-H**). The ABC_247-261_ Mimic, TT_279-296_, TT_826-845_, TT_986-1005_, TT_1058-1077_, TT_1114-1133_, and TT_1272-1284_ epitopes efficiently activated T cells *ex vivo*. Among these peptides, the ABC_247-261_ Mimic is the most potent cross-reactive T cell epitope in that it cross-reacts with all tested TT epitopes except that of TT_92-111_ (**Figures 3A-H**). The chosen TT epitopes exhibit intramolecular cross-reactivity with one another and other TT epitopes, making TT a strong immunogen (9). Also, T cells from TT peptide-immunized DR3 mice showed modest T cell IFN-γ responses to SmD peptides (**Figures 3B-H**). Immunization with the ABC_247-261_ Mimic, TT_826-845_, and TT_1058-1077_ induced cross-reactive T cells to SmD_46-75_ or SmD_61-90_ which include the SmD_66-80_ epitope (**Figures 3A-C**). These three epitopes were chosen for further investigation based on their T cell cross-reactivity with SmD_66-80_.

**Table 1:** List of T cell epitope peptides used in this study.

Name	Sequence	Reference
SMD_66-80_	RYFILPDSLPLDTLL	(9)
ABC_247-261_ Mimic	AKIIRPENIKYSACK	This work
TT_31-45_	DIYYKAFKITDRIWI	This work
TT_92-111_	VKLFNRIKNNVAGEALLDKI	(18)
TT_279-296_	DANLISIDIKNDLYEKTL	(20)
TT_506–525_	NYSLDKIIVDYNLQSKITLP	(19)
TT_826–845_	NILMQYIKANSKFIGITELK	(19)
TT_986–1005_	SIGSGWSVSLKGNNLIWTLK	(19)
TT_1058–1077_	ITGLGAIREDNNITLKLDRC	(19)
TT_1114–1133_	LRDFWGNPLRYDTEYYLIPV	(19)
TT_1272-1284_	NGQIGNDPNRDIL	(20)

TT, Tetanus toxoid. The amino acids (AAs) in the yellow background are the core AAs for epitope recognition determined by alanine substitution (9). The red AAs show which residues in ABC_247-261_ Mimic may contribute to the cross-reactivity to SMD_66-80_.

**Figure 4:**
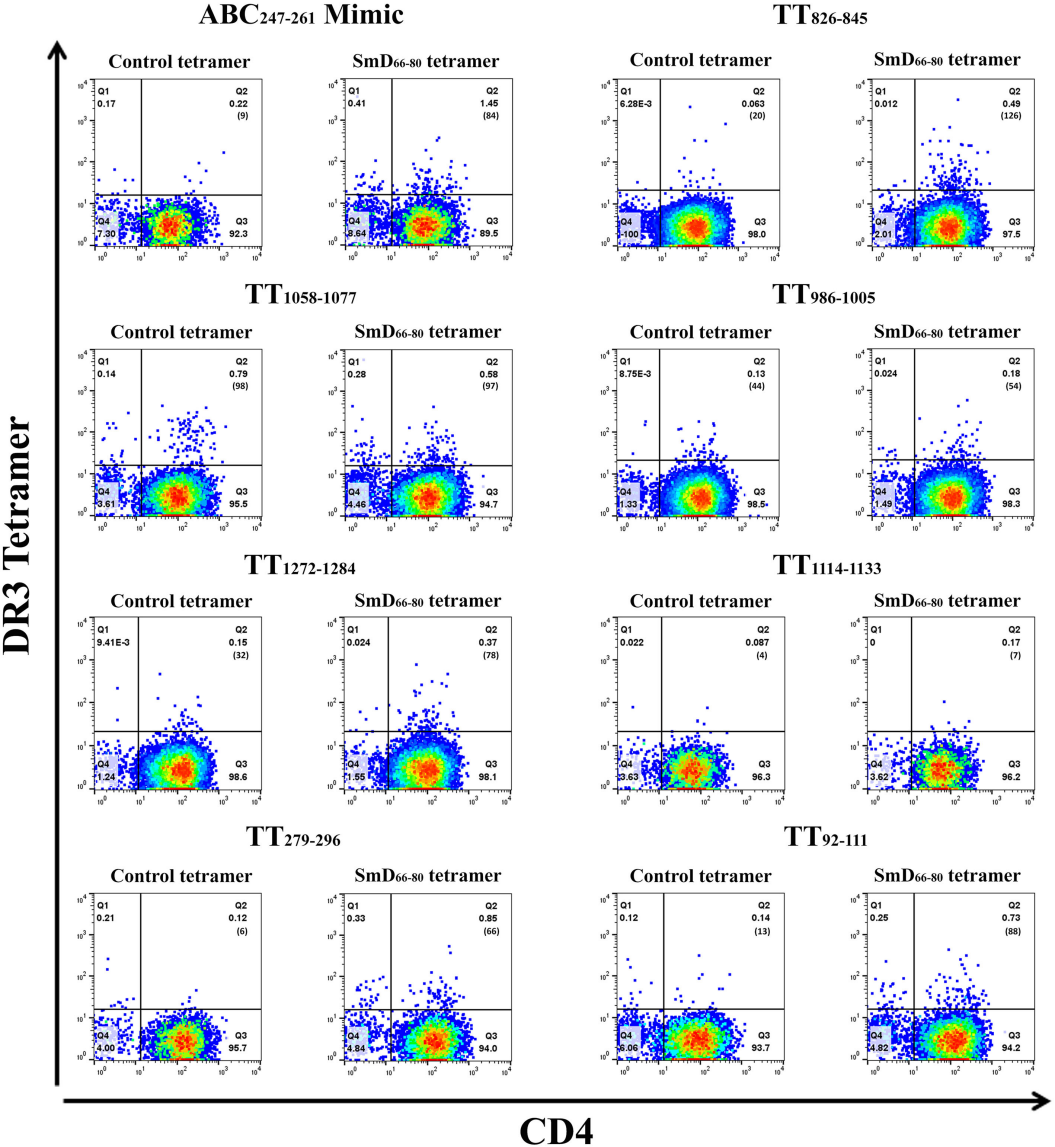
TT epitopes increase SmD_66-80_ reactive T cells *in vivo*. Peptides ABC_247-261_ Mimic, TT_826-845_, TT_1058-1077_, TT_986-1005_, TT_1272-1284_, TT_1114-1133_, TT_279-296_, and TT_92-111_ were used to immunize DR3 mice. At day 10 post immunization, the splenic CD4^+^ T cells from two or three mice were pooled together and stained with an SmD_66-80_ DR3 tetramer. The number of tetramer positive cells is shown in parentheses.

The SmD_66-80_ reactive CD4^+^ T cells induced after single epitope immunizations were assayed by SmD_66-80_ DR3 tetramer staining (**Figure 4**). The ABC_247-261_ Mimic, TT_826-845_, and TT_1058-1077_ induced SmD_66-80_ reactive CD4^+^ T cells. In the case of TT_1058-1077_, T cells from spleens of immunized mice showed staining by the control tetramer. We have no explanation for this staining. In this experiment, TT_92-111_ and TT_279-296_ induce SmD_66-80_ reactive T cells. This result differed from that in **Figure 3**, where TT_92-111_ and TT_279-296_ did not respond to SmD_61-90_ with IFN-γ secretion. This difference in results could be explained if the cross-reactive T cells induced by TT_92-111_ and TT_279-296_ are not of the Th1 subtype or do not initiate a Th1 immune response. The remaining three peptides, TT_986-1005_, TT_1114-1133_, and TT_1272-1284_ stimulation did not induce cross-reactive T cells to SmD_66-80_.

To identify the cross-reactive T cells *in vivo* due to TT exposure, we immunized DR3 mice with TT and detected the cross-reactive T cells using two DR3 tetramers with different fluorochromes. One was the SmD_66-80_ DR3 tetramer and the others were the ABC_247-261_ Mimic, TT_826-845_, and TT_1058-1077_ DR3 tetramers (**Supplementary Figure 2**). DR3 tetramer staining revealed 0.57% ABC_247-261_ Mimic cross-reactive T cells to SmD_66-80_ compared to 0.29% and 0.49% with the TT_826-845_ and TT_1058-1077_ peptides, respectively. The results showed cross-reactive T cells to SmD_66-80_ and the ABC_247-261_ Mimic are generated in DR3 mice after TT immunization as well as those cross-reactive to SmD_66-80_ and TT_826-845_ and TT_1058-1077_.

### Immunization of DR3 mice with the ABC_247-261_ Mimic generates anti-SmD autoantibodies and leads to B cell epitope spreading to other lupus-related autoantigens

**Figure 5:**
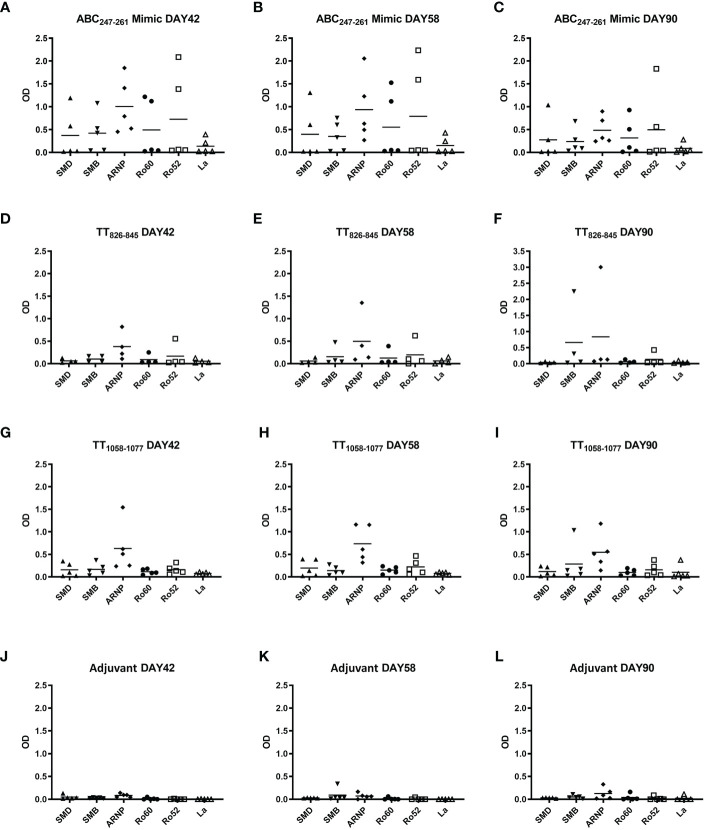
ABC_247-261_ Mimic, TT_826-845_, and TT_1058-1077_ induce autoantibodies to lupus-related autoantigens. Single T cell epitope peptides ABC_247-261_ Mimic **(A–C)**, TT_826-845_ **(D–F)**, and TT_1058-1077_ **(G–I)** were used to immunize DR3 mice. DR3 mice immunized with adjuvant only were used as controls **(J–L)**. Autoantibodies to lupus-related autoantigens were assayed in mouse sera at day 42 **(A, D, G, J)**, day 58 **(B, E, H, K)**, and day 90 **(C, F, I, L)**. Each dot indicates a single animal.

The ABC_247-261_ Mimic induced autoantibodies to SmD and other lupus-related autoantigens 42 days post immunization (**Figure 5A**). Compared to the ABC_247-261_ Mimic, peptides TT_826-845_ and TT_1058-1077_ failed to induce anti-SmD autoantibodies and induced much less B cell epitope spreading to other lupus-related autoantigens during the same time period (**Figures 5D, G**). Autoantibody titers remained detectable 58 days after DR3 mice immunizations with the ABC_247-261_ Mimic, the TT_826-845_ and the TT_1058-1077_ peptides, respectively (**Figures 5B, E, H**). The autoantibodies from the ABC_247-261_ Mimic immunizations started to decrease, while some autoantibodies from the TT_826-845_ and TT_1058-1077_ immunizations increased 90 days post immunization (**Figures 5C, F, I**). The adjuvant control showed no autoantibody production at any time point (**Figures 5J–L**).

### SLE patients have a greater percentage of ABC_247-261_ Mimic and TT_826-845_ cross-reactive T cells than healthy donors

**Figure 6:**
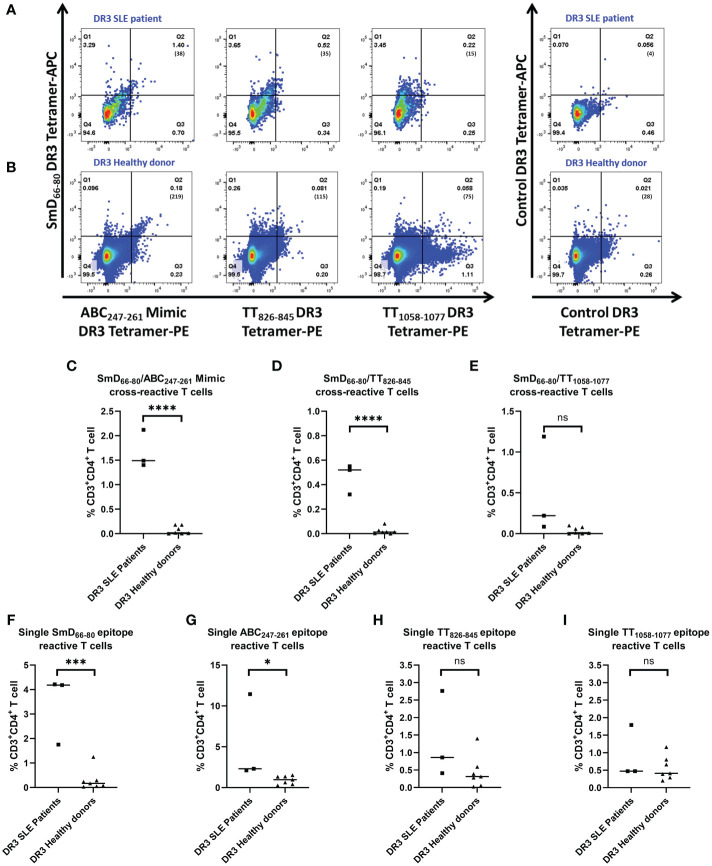
Percentages of ABC_247-261_ Mimic and TT_826-845_ cross-reactive and SmD_66-80_ and ABC_247-261_ Mimic single reactive T cells are greater in SLE patients. PBMCs were collected from DR3 SLE patients and healthy donors. They were stimulated with TT lysate for one week and stained with both SmD_66-80_ and one of three environmental epitope DR3 tetramers. Representative DR3 tetramer gating is shown for a single SLE patient **(A)** and healthy donor **(B)**. The number of tetramer positive cells is shown in parentheses. **(C–E)** Percentages of the autoimmune, cross-reactive T cells detected in SLE patients and healthy donors. **(F–I)** Percentages of the single reactive T cells against SmD_66-80_, ABC_247-261_ Mimic, TT_826-845_, and TT_1058-1077_ present in patients and healthy donors. Each dot indicates a single individual. *p < 0.05, ***p < 0.001, ****p < 0.0001 by Student’s two-tailed t-test. ns, not significant.

To support the findings from our mouse studies, we evaluated the presence of SmD_66-80_, ABC_247-261_ Mimic, TT_826-845_ and TT_1058-1077_ mono- and cross-reactive T cells in DR3 SLE patients and healthy donors. The PBMCs from both groups were stimulated with TT lysate for 5-7 days and then stained with DR3 tetramers. Representative DR3 tetramer gating is shown for a single lupus patient and healthy donor (**Figures 6A, B**). SLE patients had a greater percentage of the ABC_247-261_ Mimic and the TT_826-845_ cross-reactive T cells to SmD_66-80_ compared to healthy donors (**Figures 6C, D**). Patients also had a greater percentage of SmD_66-80_ and ABC_247-261_ Mimic mono-reactive T cells than healthy donors (**Figures 6F, G**). However, there was no significant difference in the percentages of T cells cross-reactive with TT_1058-1077_ and SmD_66-80_ (**Figure 6E**). There were no significant differences of T cells reactive only to TT_826-845_ or TT_1058-1077_ between patients and healthy donors (**Figures 6H, I**).

## Discussion

While analyzing T cells from a DR3 lupus patient, TT lysate was unexpectedly found to induce cross-reactive T cells against SmD_66-80_. This finding prompted us to examine TT epitopes and their abilities to drive autoimmune T cell responses and production of lupus-related autoantibodies. The possibility that vaccines may initiate autoimmune responses and even induce disease has been considered. There are case reports of lupus diagnoses occurring after TT immunization and lupus-related autoantibodies being generated after flu and hepatitis B virus (HBV) vaccines (21–23). These studies are limited because it is unclear whether the vaccines correlated with the production of autoantibodies and development of clinical symptoms, and they were unable to describe the details of the underlying mechanisms. To understand how TT T cell epitopes could drive autoimmune responses, we immunized humanized DR3 mice with single TT T cell epitopes. Interestingly, we found multiple intramolecular, cross-reactive T cell epitopes, a characteristic of self-antigens that makes them very antigenic (9). This explains the high level of antigenicity of TT. The ABC_247-261_ Mimic is one epitope from the same bacterium that produces TT. ABC_247-261_ Mimic reactive T cells from DR3 immunized mice are activated after priming with TT epitopes such as TT_826-845_, TT_986-1005_, TT_1058-1077_, TT_1114-1133_, and TT_1272-1284_. TT_826-845_ and TT_1058-1077_ induced autoantibodies as early as 10 days post immunization (data not shown). However, only the ABC_247-261_ Mimic induced anti-SmD autoantibodies and B cell epitope spreading to other lupus-related autoantigens months after immunization. These results suggest the ABC_247-261_ Mimic T cell epitope is a long-term driver of autoimmunity.

To analyze antigen-specific T cells in our studies, we used HLA tetramers: powerful tools to monitor T cells with specific epitope reactivity. Cross-reactive T cells that can be activated by both autoantigens and non-autoantigens have been detected in several autoimmune diseases in addition to lupus e.g. rheumatoid arthritis, antiphospholipid syndrome, celiac disease, autoimmune encephalomyelitis, multiple sclerosis and diabetes (24–33). In a mouse model of SLE, mouse H-2 tetramers were used to probe for U1-70_131-150_ (another lupus-related autoantigen epitope) reactive T cells. In the autoimmune-prone MRL/lpr mice, U1-70 reactive T cells increase with disease severity (34). However, the U1-70 study is limited by its use of mouse H-2 tetramers in a mouse system. In our experiments, we studied the SmD autoantigen epitope as well as the cross-reactive TT epitopes. Additionally, we employed humanized DR3 mice and human samples from DR3 lupus patients and healthy donors.

The phenotypes observed in mice are not directly attributable to humans because their immune systems are not the same (35). Thus, we also utilized human specimens in our studies to validate observations from our mouse model. Similar to our mouse studies, we detected T cells cross-reactive between SmD_66-80_ and *Clostridium* tetani epitopes in human PBMCs. Using the DR3 tetramers, it is not possible to rule out that within the population of cross-reactive T cells there are no dual T cell receptor (TCR) T cells present (36). However, given dual TCR T cells make up a small fraction of the entire population of T cells, it is more than likely that most of the T cells we identified are cross-reactive T cells. Further experimentation could be used to identify the specific TCRs of these cells. In addition, the percentages of cross-reactive T cells in our samples may be higher than what we detected due to competition between the two tetramers for binding to the cells (**Supplementary Figure 3**).

To our knowledge, there are no reports about TT epitopes that cross-react with self-antigens. In two studies of autoimmune diseases using tetramers, small percentages of cross-reactive T cells to microbial and self-epitopes were detected, similar to those detected in our study (30, 37).

Compared to healthy donors, lupus patients have greater percentages of SmD_66-80_ and ABC_247-261_ Mimic mono-reactive T cells, but not single TT_826-845_ and TT_1058-1077_ reactive T cells. This result is congruent with clinical findings that there is no difference in anti-TT titers in SLE patients and healthy donors after TT vaccination (38–40). But the differences in the percentages of cross-reactive T cells between lupus patients and healthy donors gives us insight into the activation of autoimmune T cells in these patients. As they encounter innumerable environmental mimics of self-antigens, autoimmune, cross-reactive T cells accumulate. Following accumulation, these cells proliferate and activate autoimmune, cross-reactive B cells, leading to autoantibody production and the breaking of self-tolerance. The autoimmune SmD_66-80_ T cells in lupus patients are not biased toward IL-17a expression (**Supplementary Figure 4**), indicating that these autoimmune CD4^+^ T cells may not only express IL-17a, but a mixture of other cytokines.

We are interested in investigating the abilities of environmental peptides to induce autoimmune T cells and autoantibody generation. Our hypothesis is that autoantibodies and autoreactive T cells are generated by the activation of T cells cross-reactive with autoantigens and environmental antigens (e.g. those present in bacteria, viruses, fungi, etc.) in an HLA-DR restricted manner. Molecular mimicry is the underlying mechanism of induction of autoantibodies by the ABC_247-261_ Mimic, TT_826-845_, and TT_1058-1077_ due to cross-reactivity to SmD_66-80_. Removing these autoimmune, cross-reactive T cell epitopes from vaccines may benefit individuals susceptible to developing lupus or other autoimmune diseases.

In this investigation, the importance of HLA-DR3 in the pathogenesis of SLE is emphasized. However, the genetics of lupus is very complex. The female mice of NZM2328.DR3.AE0 mice have more anti-SmD antibodies and more severe nephritis with early mortality (41). No anti-SmD antibodies were detected in SJL.DR3.AE0 mice immunized with ABC_247-261_ Mimic (Zhao, unpublished data), although significantly increased autoantibody to SmD was detected in the presented B10.DR3.AE0 immunization (**Figures 5A-C**). These data highlight the importance of non-major histocompatibility complex genes which may be protective and prevent autoimmunity. Thus, although TT and the ABC_247-261_ Mimic may induce autoantibodies, only in rare instances, diseases such as SLE and related disorders result.

## Data availability statement

The original contributions presented in the study are included in the article/**Supplementary Material**. Further inquiries can be directed to the corresponding author.

## Ethics statement

The animal study was reviewed and approved by the Animal Care and Use Committees at the University of Virginia.

## Author contributions

ZZ, WWK, and SMF designed the research. ZZ, ANA, and CCK carried out the experiments. All the authors were involved in the analysis and interpretation of the data. ZZ, FG, ANA, and SMF wrote the manuscript. ZZ and SMF had full access to all the data and are responsible for the integrity and accuracy of the data analysis. All authors contributed to the article and approved the submitted version.

## Funding

This work was supported in part by National Institutes of Health Grant R01 139673 (to SMF) and ANA was supported by training grants 5T32DK 072922 (PI: Mark Okusa) and 1TL1DK 132771 (PI: Portilla Didier).

## Conflict of interest

The authors declare that the research was conducted in the absence of any commercial or financial relationships that could be construed as a potential conflict of interest.

## Publisher’s note

All claims expressed in this article are solely those of the authors and do not necessarily represent those of their affiliated organizations, or those of the publisher, the editors and the reviewers. Any product that may be evaluated in this article, or claim that may be made by its manufacturer, is not guaranteed or endorsed by the publisher.
