# Supplementary material for: HLA-DR3 restricted environmental epitopes from the bacterium Clostridium tetani have T cell cross-reactivity to the SLE-related autoantigen SmD
Source: Front Immunol. 2022 Oct 31;13:928374. doi: 10.3389/fimmu.2022.928374 (PMC9659850; doi:10.3389/fimmu.2022.928374)
Supplement: Supplementary file 1 [file DataSheet_1.docx]

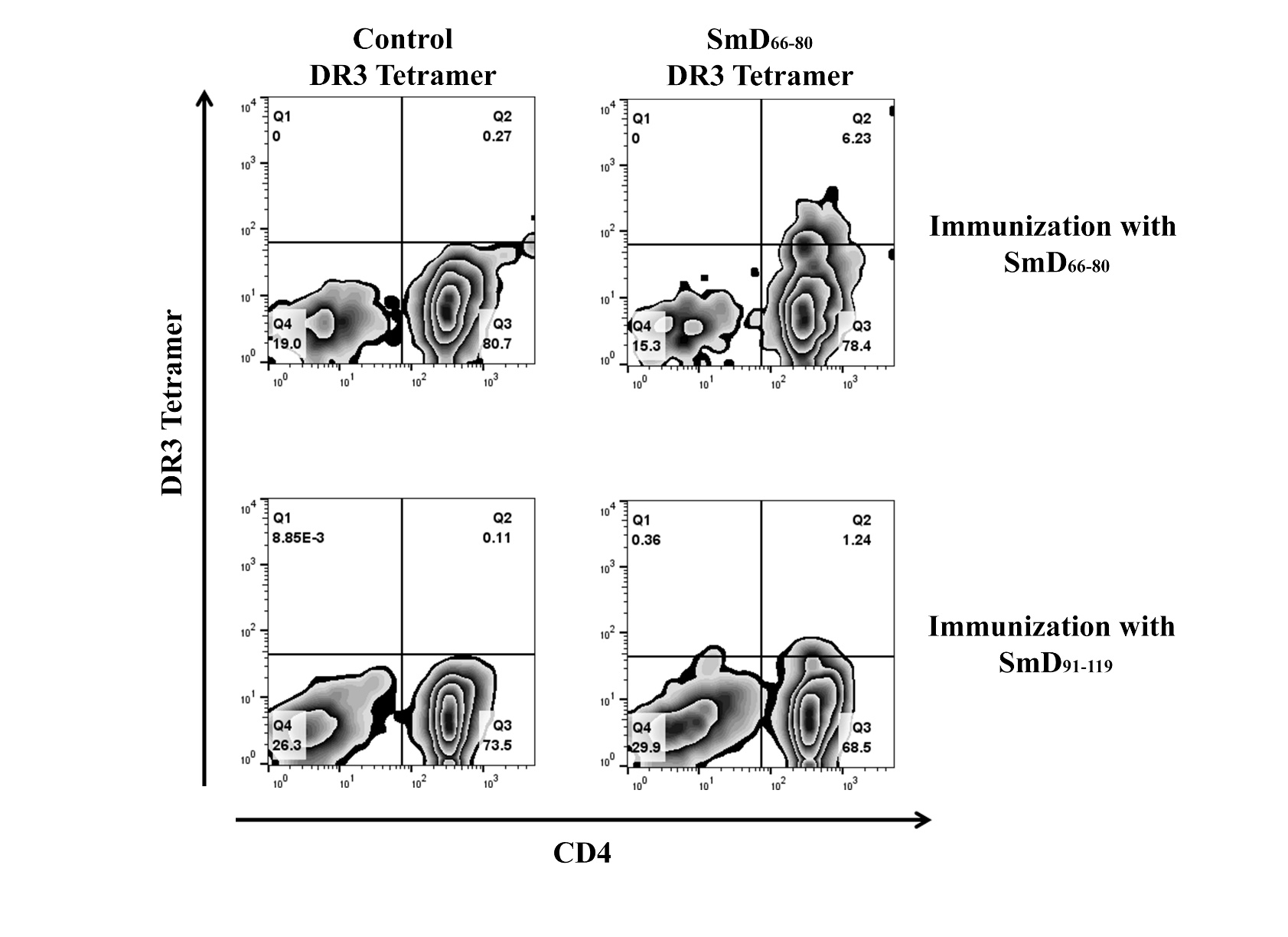


**Supplementary Figure 1 | The SmD_66-80_ DR3 tetramer specifically stains SmD_66-80_ reactive CD4^+^ T cells.** The SmD_66-80_ DR3 tetramer was used to stain splenic cells from SmD_66-80_ immunized DR3 mice (top). The splenic cells from SmD_91-119_ immunized mice were used as a control (bottom).


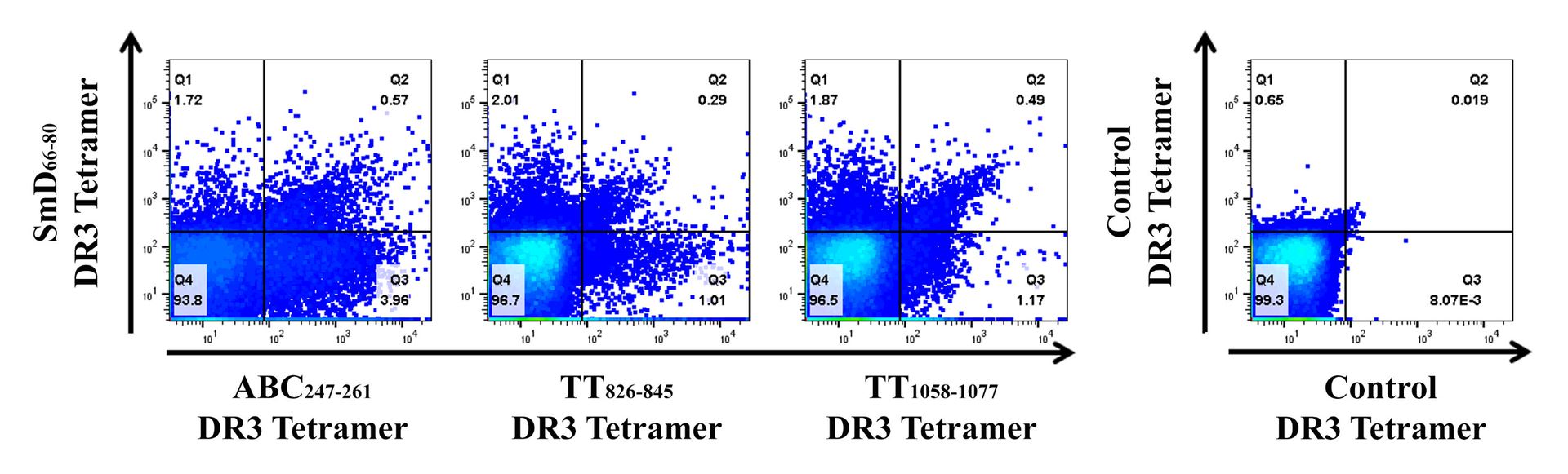


**Supplementary Figure 2 | DR3 mice have cross-reactive CD4^+^ T cells to SmD_66-80_ and three *Clostridium tetani* epitopes after TT immunization.** Splenic cells from TT immunized DR3 mice were stained with two DR3 tetramers with different epitopes and fluorochromes.


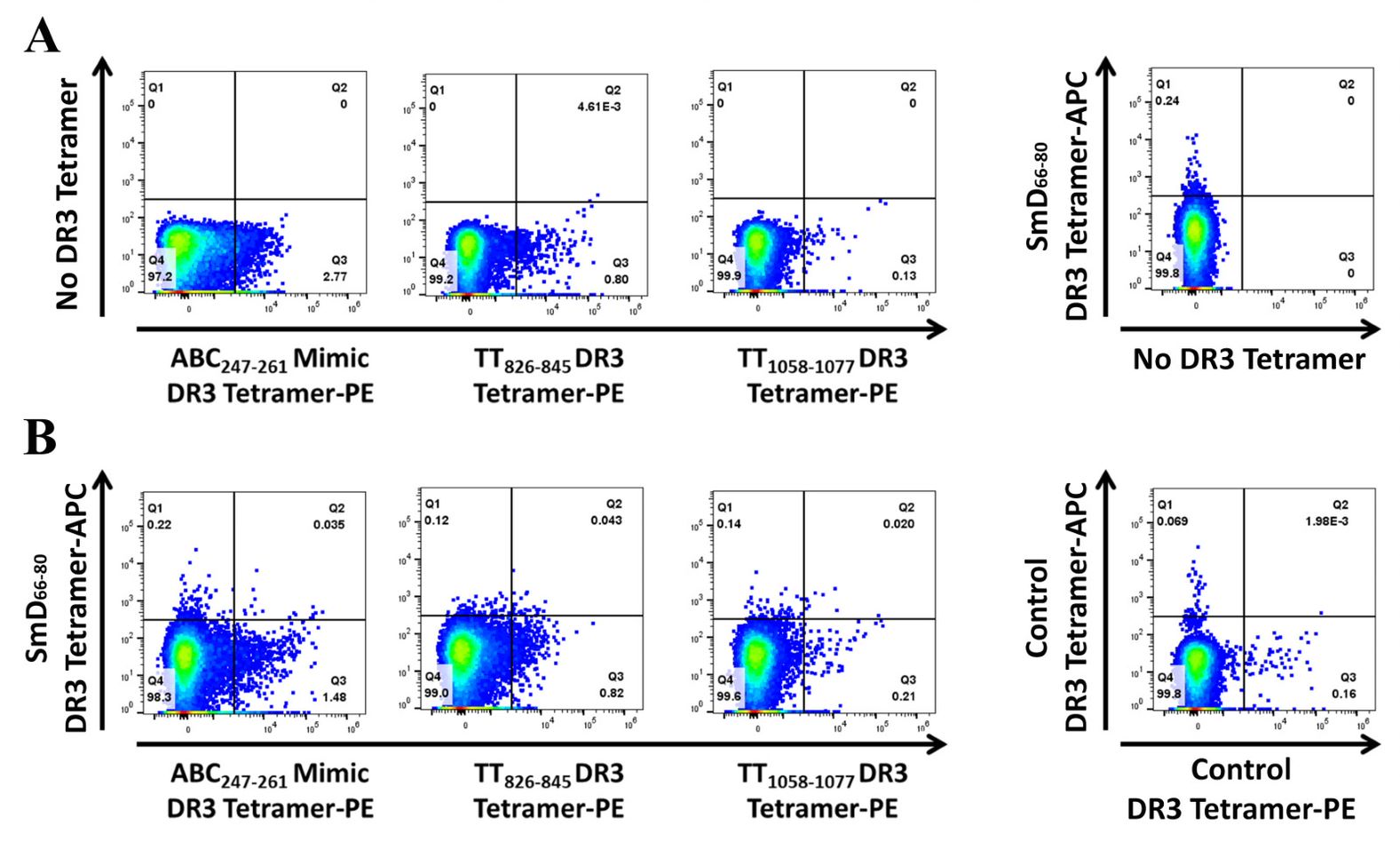


**Supplementary Figure 3 | DR3 tetramers compete for binding to cells when used for dual staining of human PBMCs.** When human PBMCs are dual stained with DR3 tetramers, there is a decrease in the percentage of SmD_66-80_ and ABC_247-261_ Mimic positively stained cells, compared to single DR3 tetramer staining. Due to the competition of the tetramers, the percentage of cross-reactive T cells in our samples may be higher than what was detected.


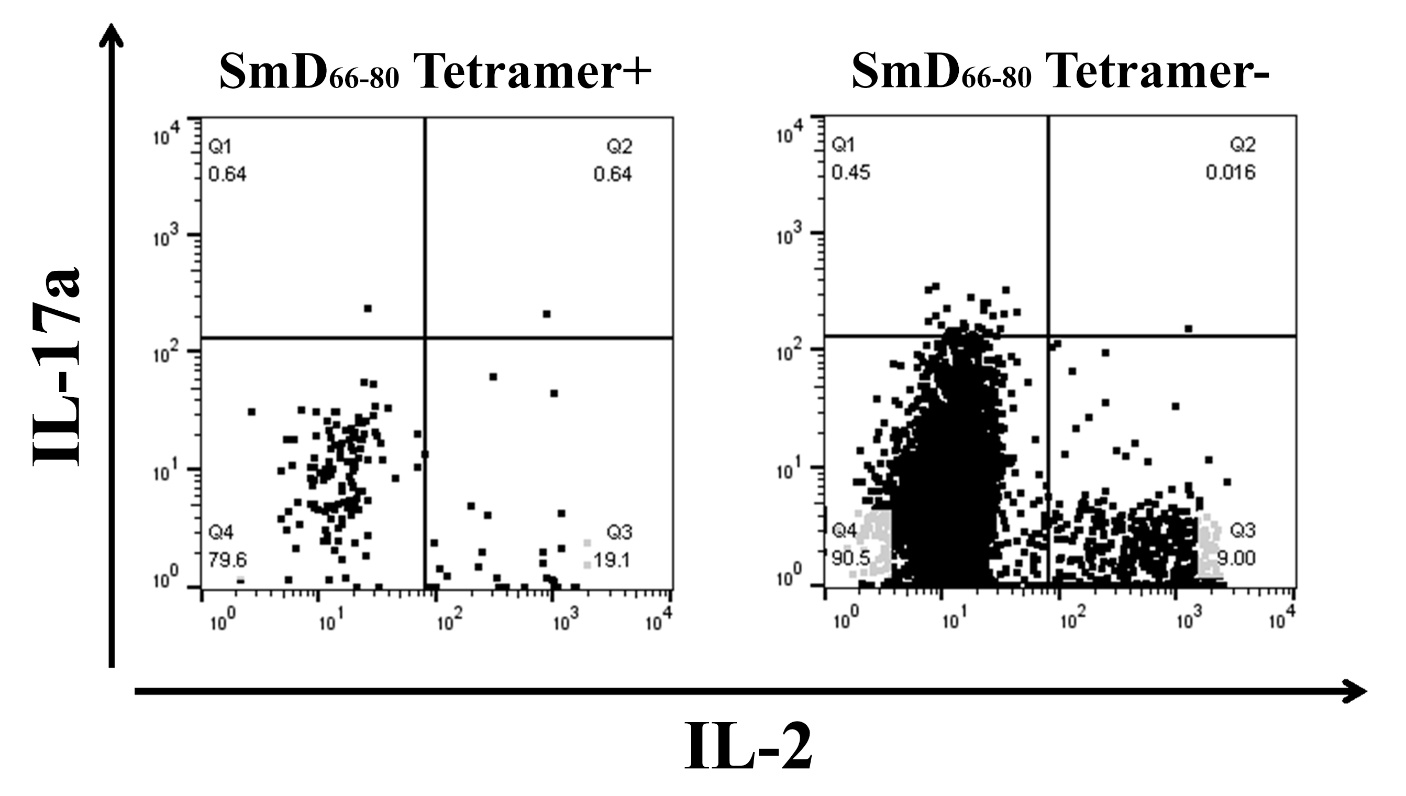


**Supplementary Figure 4 | SmD_66-80_ reactive CD4^+^ T cells from one DR3 positive SLE patient produce IL-2 and other cytokines in addition to IL-17a.** The PBMCs from a DR3 SLE patient were stimulated with phorbol 12-myristate 13-acetate (PMA) for 6 hours and then stained with and without the SmD_66-80_ DR3 tetramer and intracellular antibodies to IL-2 and IL-17a.
